# A comparison of organs at risk doses in GYN intracavitary brachytherapy for different tandem lengths and bladder volumes

**DOI:** 10.1120/jacmp.v17i3.5584

**Published:** 2016-05-08

**Authors:** Zahra Siavashpour, Mahmoud Reza Aghamiri, Ramin Jaberi, Naser ZareAkha, Hamid Reza Dehghan Manshadi, Christian Kirisits, Mahbod Sedaghat

**Affiliations:** ^1^ Department of Medical Radiation Engineering Shahid Beheshti University Tehran Islamic Republic of Iran; ^2^ Department of Radiotherapy Tehran University of Medical Science Tehran Iran; ^3^ Department of Brachytherapy Pars Hospital Tehran Iran; ^4^ Department of Radiotherapy Hafte Tir Hospital Tehran Iran; ^5^ Department of Radiotherapy and Oncology Comprehensive Cancer Center, Medical University of Vienna Austria; ^6^ Centre intégré de cancérologie de la Montérégie (CICM) Charles LeMoyne Hospital Greenfield Park QC Canada

**Keywords:** tandem length, cervical cancer, brachytherapy

## Abstract

The purpose of this study was to investigate the concurrent effects of tandem length and bladder volume on dose to pelvic organs at risk (OARs) in HDR intracavitary brachytherapy treatment of cervical cancer. Twenty patients with locally advanced cervical cancer were selected for brachytherapy using Rotterdam applicators. The patients were CT scanned twice with empty and full bladder. Two treatment plans were prepared on each of the image sets. Patients were categorized into two groups; those treated with a tandem length of 4 cm or smaller (T≤4 cm) and those with tandem length larger than 4 cm (T>4 cm). Only one tandem tip angle of 30° was studied. Dose‐volume histograms (DVHs) of OARs were calculated and compared. Bladder dose was significantly affected by both bladder volume and tandem physical length for T≤4 cm. This was reflected on the values obtained for $D_{2{\text{cm}}^{3}}$, $D_{1{\text{cm}}^{3}}$, and $D_{0.1{\text{cm}}^{3}}$ for both empty and full bladder cases. When T>4 cm, no correlation could be established between variations in bladder dose and bladder volume. Rectum dose was generally lower when the bladder was empty and T>4 cm. Dose to sigmoid was increased when T>4 cm; this increase was larger when the bladder was full. Our results suggest that, for tandems longer than 4 cm, keeping the bladder empty may reduce the dose to rectum and sigmoid. This is contrary to cases where a shorter than 4 cm tandem is used in which a full bladder (about 50–120 cm^3^) tends to result in a lower dose to rectum and sigmoid. Attention should be given to doses to sigmoid with long tandem lengths, as a larger tandem generally results in a larger dose to sigmoid.

PACS number(s): 87.53.Jw

## I. INTRODUCTION

For patients suffering from locally advanced cervical cancer, brachytherapy is an essential component of radiochemotherapy.^(1)^ The use of three‐dimensional (3D) treatment planning and high‐dose‐rate (HDR) brachytherapy is becoming a general modality for the treatment of these patients.

Three‐dimensional (3D) treatment planning relies on three dimensionally reconstructed image sets, generally obtained by either CT or magnetic resonance scanners. Such images are used in image‐guided brachytherapy (IGBT) to achieve higher dose conformity to the target and better organ at risk (OAR) preservation. This image‐guided approach theoretically ‘widens up’ what has been coined as the ‘therapeutic window’ of the treatment.^(2)^

The anatomy and topography of bladder has a substantial influence on bladder dose values in cervical cancer brachytherapy.^3^, ^4^, ^5^, ^6^, ^7^, ^8^ In order to reduce the dose to small intestine in gynecological (GYN) brachytherapy, treating the patient with a refilled bladder is often practiced.^(2)^ We also follow a protocol in our center (explained further in the text) in which the patient's bladder is refilled before the treatment. In the literature, the effect of bladder volume on dose to OARs has been studied.^3^, ^4^, ^5^, ^6^ We chose to investigate how keeping the bladder full during our treatments affects the dose to the bladder and other OARs, namely rectum and sigmoid. Intrauterine tandem length and tip angle are selected by the clinical radiotherapy oncologist depending on patients' anatomy, especially the uterus channel length and malignancy extension. We also hypothesized that tandem physical length could notably influence the dose to OARs and chose to concurrently study this effect combined with the bladder volume effect. We did not find similar studies to this hypothesis in the literature.

## II. MATERIALS AND METHODS

### A. Case selection

Twenty patients suffering from locally advanced cervical cancer were selected over a period of 18 months, between June 2012 and November 2013. The patients had complete or partial response to 3D conformal external radiotherapy (EBRT) with a prescribed dose of 45 or 50 Gy over a period of five to six weeks and were consequently nominated for brachytherapy. The patients also received concomitant weekly doses of $50\;\text{mg}/m^{2}$ cisplatin‐based chemotherapy. As no parametrial invasion was observed by magnetic resonance imaging (MRI) at the time of brachytherapy treatment, no needle insertion was prescribed for these patients; they were therefore treated only with tandem and ovoid applicators.

Patients had different FIGO (Federation of Gynecology and Obstetrics) stages of IB1, IB2, and IIB.^(9)^ Patients were informed about the study and their consent was obtained; ethical rules and considerations were observed throughout the study.

### B. Applicator insertion

Patients were treated under general or regional anesthesia with a high‐dose‐rate (HDR) Flexitron afterloader equipped with an Ir‐192 Flexisource (Elekta AB, Stockholm, Sweden) at the brachytherapy department of Atieh Hospital, Tehran, Iran. Rotterdam tandem‐ovoid applicators (Elekta AB) were used for the treatments.^(10)^ A number of tandem applicators of 3, 4, 5, and 7 cm length were available for the treatments; tandem tip angle of 30° was suited for the uterus in all patients. Ovoid caps of 2 or 3 cm diameter were used appropriately.

A Foley catheter was inserted into each patient's bladder. The bladder balloon was filled with 1 cm^3^ of Meglumine compound mixed with 6 cm^3^ of normal saline. After patient recovery and applicator fixation, CT scan was performed. Bladder volume was controlled such that the bladder was either empty or filled with 120 cm^3^ of normal saline. When empty bladder was required, the patient was duly instructed to discharge the bladder and care was taken to keep the bladder volume as low as possible.

### C. Imaging protocols

Patients were placed in supine position on a flat backboard on the CT scanner table (Somatom DR, Siemens Healthcare, Forchheim, Germany). They were CT scanned with full bladder after a solution of 120 cm^3^ of normal saline and Meglumine compound was injected into their bladder through the Foley catheter and its lumen was clamped. The scan was repeated once again when the patients emptied their bladders. The whole pelvis was scanned with slices of 3 mm thickness. Scanning with full and empty bladder was performed in just one fraction of the treatment for each patient. The bladder was refilled with the same volume of normal saline (120 cm^3^) just before the treatment to reproduce imaging conditions.

### D. Contouring, treatment planning, and treatment protocol

Treatment plans were created with Flexiplan 3D treatment planning system (TPS), version 2.6 (Isodose Control B.V., Ede, The Netherlands), using a TG‐43‐based calculation algorithm without inhomogeneity corrections.^(11)^ CT images were imported into the TPS. The same radiation oncologist contoured intermediate‐ and high‐risk clinical target volumes (${\text{CTV}}_{\text{IR}}$ and ${\text{CTV}}_{\text{HR}}$) on both empty and full bladder CT image sets of each patient following GEC‐ESTRO recommendations.^12^, ^13^ This was to minimize uncertainties in contouring which could have a negative impact on our results. Organ volumes were inspected on both CT image sets before and after the treatment and no significant variations were detected. Bladder, rectum, and sigmoid were also contoured as OARs. The proper applicator model was selected from the TPS library and positioned over the applicator profile on CT images using the TPS three‐point positioning algorithm; it was manually adjusted for best fit.

Contouring and forward‐treatment planning were performed separately on each of the two image sets with the same applicator type and treatment planning aim. Each clinical case was considered as an individual plan; all dose constraints for OARs were matched as closely as possible between the two plans. The dose encompassing 90% of the target volume ($D_{90}$) was carefully adjusted to be the same in both bladder conditions. In some instances $D_{90}$ was decreased in one of the two treatments plans to avoid over exposing OARs. Dose per fraction was calculated using a biologically equivalent dose of 2 Gy per fraction (EQD2) and linear‐quadratic model with α/β=10 Gy for CTVs and α/β=3 Gy for OARs.^(2)^ GEC‐ESTRO guidelines for contouring and dose constrains on normal tissues and CTVs were observed in treatment planning.^12^, ^13^, ^14^ The treatment aim was to deliver a minimum total dose of 80‐90 Gy to $D_{90}$ of the ${\text{CTV}}_{\text{HR}}$ (EQD2). The prescribed dose to ${\text{CTV}}_{\text{HR}}$ included 45‐50 Gy from EBRT plus 30–40 Gy EQD2 from brachytherapy in 3 to 4 fractions. The ${\text{CTV}}_{\text{HR}}$ volume was between 15‐19 cm^3^ in all cases. ${\text{CTV}}_{\text{IR}}$ is the volume of tumor at the time of diagnosis or ${\text{CTV}}_{\text{HR}}$ plus GEC‐ESTRO‐recommended margins; we aimed at delivering a dose of 70 Gy EQD2 as $D_{90}$ to ${\text{CTV}}_{\text{IR}}$.^12^, ^13^ It follows that the extension of the ${\text{CTV}}_{\text{IR}}$ determined the activation length of the tandem. In just one instance we inevitably activated a shorter length of the tandem to preserve OARs. Therefore, tandem length was equal to the activation length in all but one of our plans. Dose constrains were defined as a maximum of 70 and 80 Gy to $D_{2{\text{cm}}^{3}}$ of the rectum and bladder, respectively.^(13)^ The treatment protocol described above was our standard institutional protocol and was applied unaltered to all patients in this study.

### E. Classification

Patients were categorized into two groups: those treated with a tandem length of 4 cm or smaller (T≤4 cm) and those with a tandem length larger than 4 cm (T>4 cm). The two groups contained 6 and 14 patients, respectively. Treatment plans were also categorized into full bladder ($B_{\text{Full}}$) and empty bladder ($B_{\text{Empty}}$) categories.

### F. Data analysis

Dose‐volume histograms (DVHs) were calculated for all cases in Flexiplan environment. Statistical analysis including paired samples *t*‐test and multivariate general linear model (GLM) test were performed with SPSS (SPSS Statistics for Windows, Version 17.0; Chicago, SPSS Inc.), using confidence intervals (CI) of 95.0% (equivalent to significance level of 0.05) throughout.

To validate our results, we checked whether our statistical data for the *t*‐test and GLM had a normal probability distribution using Kolmogorov‐Smirnov (KS) analysis; also homogeneity of the variances of the GLM was validated with Levene's test.

A MATLAB (The MathWorks, Natick, MA) code was developed in‐house to automatically calculate distances between organ structures. All organ contours were exported in DICOM format into the code and the minimum distance between each organ and the CTV was determined for both $B_{\text{Full}}$ and $B_{\text{Empty}}$ treatment plans. The distances were calculated for each patient and averaged for each organ over all patients.

## III. RESULTS

For all tandem lengths, an average difference of 0.3%±2.1% was obtained for ${\text{CTV}}_{\text{HR}}$ volume between full and empty bladder conditions ($B_{\text{Full}}$ and $B_{\text{Empty}}$), which was insignificant. The $D_{90}$ for ${\text{CTV}}_{\text{HR}}$ in full bladder cases was systematically only 2% smaller than empty bladder cases, which was also insignificant; therefore, our dose prescription to ${\text{CTV}}_{\text{HR}}$ in both $B_{\text{Full}}$ and $B_{\text{Empty}}$ treatment plans was fairly similar.

The mean volumes (±1 SD (σ)) of empty and full bladder were $59.7\pm 18.8\;{\text{cm}}^{3}$ and $177.4\pm 44.6\;{\text{cm}}^{3}$, respectively. For rectum and sigmoid, the mean (±1σ) volume differences between $B_{\text{Full}}$ and $B_{\text{Empty}}$ treatment plans were 0.5%±5.9% and 0.6%±4.6%, respectively. These small volume changes between the two bladder conditions were expected as the two CT scans were obtained subsequently with a short time delay.

Minor variations in the time of delivery were observed between treatment plans with full and empty bladder, yet without statistical significance (p‐value of 0.726).

### A. Effect of bladder volume on dose to OARs

Table 1 shows a comparison of bladder, rectum, and sigmoid dose volume parameters of the two $B_{\text{Full}}$ and $B_{\text{Empty}}$ treatments plans. The distance between the organs and ${\text{CTV}}_{\text{HR}}$ was calculated and the mean distance difference between the two bladder conditions averaged over all patients is depicted in the table. Notable differences in bladder volume and dose are apparent, as was expected. Rectum and sigmoid received a slightly higher dose when the bladder was full, but the differences were not statistically significant. The change in $D_{2{\text{cm}}^{3}}$ of OARs between full and empty bladder groups was calculated as $\left(\text{Dose}(B_{\text{Full}})-\text{Dose}\;(B_{\text{Empty}})\right)$; the values obtained in EQD2 per fraction were 1.3±2.8 Gy, 0.1±1.3 Gy, and 0.4±2.1 Gy for bladder, rectum, and sigmoid, respectively. Minimum distances between OARs and ${\text{CTV}}_{\text{HR}}$ were smaller in $B_{\text{Full}}$ treatments plans, but this was inconsequential compared to $B_{\text{Empty}}$ plans.

**Table 1:** Comparison of OARs DVH parameters and minimum distance to ${\text{CTV}}_{\text{HR}}$ for each organ between the full and the empty bladder groups, regardless of tandem length. P‐values in parentheses are paired samples statistical test results.

	*Mean* $\left((B_{\text{Full}})-(B_{\text{Empty}})\right)$ *DVH Parameters* ±1σ								
	*% of Planning Aim Dose* ±1σ *(p‐value)*						*% of OAR Volume* ±1σ *(p‐value)*		*Min. Dis.* ^a^ *to* ${\text{CTV}}_{\text{HR}}$ *(mm)*
	$D_{2{\text{cm}}^{3}}$	$D_{0.1{\text{cm}}^{3}}$	$D_{1{\text{cm}}^{3}}$	$D_{10}$	$D_{30}$	$D_{50}$	$V_{100}$	$V_{50}$	
Bladder	8.8±18.5 (0.047)	17.1±31.1 (0.024)	9.9±20.1 (0.047)	−6.6±12.6 (0.030)	−7.7±9.3 (0.001)	−7.1±7.6 (0.001)	−0.8±2.9 (0.232)	−6.7±11.2 (0.015)	−2.8±4.4
Rectum	0.7±8.2 (0.726)	2.0±13.4 (0.533)	–	0.3±6.6 (0.853)	0.7±5.0 (0.521)	1.2±3.7 (0.193)	–	–	−1.3±1.9
Sigmoid	0.3±11.5 (0.901)	5.3±38.4 (0.543)	–	0.8±11.2 (0.744)	0.8±7.3 (0.641)	0.5±5.4 (0.707)	–	–	−0.2±1.4

a Min. Dis. is the mean difference of minimum distance between the organ and ${\text{CTV}}_{\text{HR}}$ in the two bladder conditions, calculated as $\Sigma P_{i}/n$; where $P_{i}=\text{Distance}(B_{\text{full}})-\text{Distance}(B_{\text{Empty}})$ and *n* is the number of samples (i.e., 20).

### B. Effect of tandem length and bladder volume on the OARs received dose

#### B.1 Bladder

**Figure 1:**
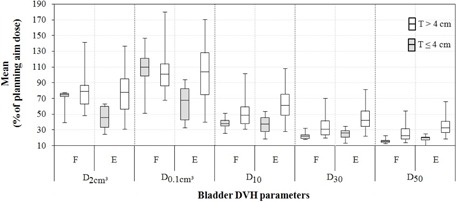
Box plot representation of mean variations of dose‐volume parameters for the bladder; the values are averages of the corresponding parameter over all cases under one of the four categories of tandem length and bladder condition calculated in Flexiplan (T=tandem length, E=empty bladder, and F=full bladder).

Figure 1 is a graphical representation of five dose‐volume parameters (i.e., $D_{2{\text{cm}}^{3}}$, $D_{0.1{\text{cm}}^{3}}$, $D_{10},D_{30}$, and $D_{50}$) for the two tandem length groups and bladder conditions. Note that there were 14 insertions (70% of the cases) with a tandem length longer, and six (30% of the cases) with a length shorter than or equal to 4 cm. The fractional volume of bladder receiving 100% of the prescribed dose ($V_{100}$) was almost identical in all treatment plans (not shown).

**Figure 2:**
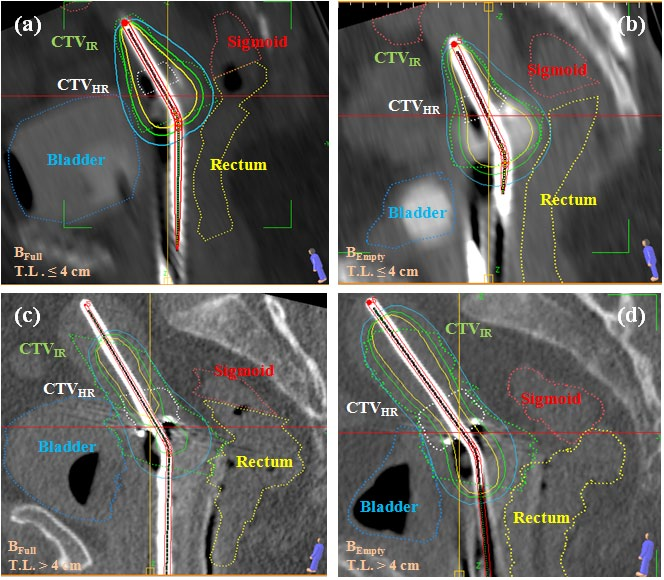
Contoured CT scan examples of two cervical cancer patients with inserted applicators: (a) and (b) tandem length<4 cm, and (c) and (d) tandem length<4 cm. Note: full bladder (a) and (c); empty bladder (b) and (d). Tandem length≠activation length in (c) and (d). (T.L.=tandem length, $B_{\text{Empty}}=\text{empty bladder}$, and $B_{\text{Full}}=\text{full bladder}$).

The figure indicates that:

1. When T≤4 cm, notably smaller dose values were obtained for $D_{2{\text{cm}}^{3}}$ and $D_{0.1{\text{cm}}^{3}}$ with an empty bladder, while all other dose volume parameters were almost identical in both bladder conditions.
2. When T>4 cm, almost identical values were obtained for $D_{2{\text{cm}}^{3}}$ and $D_{0.1{\text{cm}}^{3}}$ for both full and empty bladder conditions, while all other dose‐volume parameters were smaller for the full bladder (note the medians of each box). Figure 2 shows sagittal views of the tandem length in four different treatment plans with full and empty bladders. The figure visually represents a sample of cases prepared, examined, and analyzed for this study. The figure contains the only case in our study where a shorter length (by about 9 mm) than the tandem physical length was activated to avoid overexposing sigmoid which happened to fall extremely close to the tandem.

#### B.2 Rectum and sigmoid

2, 3 summarize mean values of dose‐volume parameters for rectum and sigmoid, respectively. Data presented in these tables were calculated as percentage (%) of planning aim dose in the TPS. For example, if the aim dose per fraction for a plan was 8 Gy and $D_{2{\text{cm}}^{3}}$ of an OAR was 5.2 Gy, then the percentage of planning aim dose for that OAR would become 65% (i.e., (5.2×8)×100%=65%). In the tables, GLM test results are presented as significance of the differences between classified groups. The tables also contain mean minimum distance of each organ to ${\text{CTV}}_{\text{HR}}$ for the $B_{\text{Full}}$ and $B_{\text{Empty}}$ treatment plans.

As observed in Table 2, the effect of tandem length on rectum dose in full and empty bladder groups was not significant (p‐value >0.05); nevertheless, the dose to rectum decreased for tandem lengths longer than 4 cm when the bladder was empty. Contrary to this, for tandems shorter than 4 cm, the dose to rectum was decreased when the bladder was full. These decreases on the dose‐volume parameter $D_{2{\text{cm}}^{3}}$ were about 3.2% and 7.6% of the planning aim dose for the aforesaid two tandem length categories, respectively.

For the sigmoid (see Table 3), statistical analysis revealed no significant differences between all groups. However, the mean dose to sigmoid was higher for T>4 cm, in both empty and full bladder groups; note that the dose to sigmoid increases more when the bladder is full. Therefore, with a discharged bladder sigmoid tends to receive a smaller dose increase when T>4 cm.

When T≤4 cm, the mean difference in $D_{2{\text{cm}}^{3}}$ for bladder, expressed in EQD2, between the full and empty bladder conditions (i.e., Mean ($B_{\text{Full}}$ ‐ $B_{\text{Empty}}$) ±1σ (Gy)) was 4.2±2.4 Gy per fraction. These dose differences for rectum and sigmoid were −0.8±0.52 Gy and −0.4±1.0 Gy EQD2 per fraction, respectively. Assuming 3 to 4 fractions for each treatment, an empty bladder would receive a substantially lower dose of about 12 Gy EQD2 over the course of the treatment. For T>4 cm, these results were 2.2±1.1 Gy, 0.46±1.36 Gy, and 0.69±2.4 Gy EQD2 per fraction, for bladder, rectum, and sigmoid, respectively.

**Table 2:** Comparison of rectum dose and minimum distance to ${\text{CTV}}_{\text{HR}}$ in full and empty bladder groups for different tandem lengths. P‐values are multivariate statistical test results.

	*T. L. (cm)*	*Mean of Rectum DVH Parameters (percentage of planning aim dose)* ±1σ					*Min. Dis.* ^a^ *to* ${\text{CTV}}_{\text{HR}}$ *(mm)*
		$D_{2{\text{cm}}^{3}}$	$D_{0.1{\text{cm}}^{3}}$	$D_{10}$	$D_{30}$	$D_{50}$	
$B_{\text{Full}}$	≤4	66.6±6.9	90.5±13.2	56.9±9.5	39.4±8.4	30.4±7.1	8.1
	>4	68.7±8.1	93.4±11.9	56.1±7.3	39.6±6.2	31.0±5.6	6.4
$B_{\text{Empty}}$	≤4	72.1±6.3	97.1±8.8	61.0±9.2	41.1±8.8	30.2±7.7	7.8
	>4	66.5±9.2	89.3±13.5	54.7±6.8	38.4±6.1	29.9±5.5	9
p‐value		0.186	0.277	0.171	0.516	0.904	0.404

a Min. Dis. is the mean of minimum distance between the organ and ${\text{CTV}}_{\text{HR}}$, calculated as $\Sigma P_{i}/n$; where $P_{i}$ is the distance and *n* is the number of samples.

T.L.=tandem length

**Table 3:** Comparison of sigmoid dose and minimum distance to ${\text{CTV}}_{\text{HR}}$ in full and empty bladder groups for different tandem lengths. P‐values are multivariate statistical test results.

	*T. L. (cm)*	*Mean of Sigmoid DVH Parameters (percentage of planning aim dose)* ±1σ					*Min. Dis.* ^a^ *to* ${\text{CTV}}_{\text{HR}}$ *(mm)*
		$D_{2{\text{cm}}^{3}}$	$D_{0.1{\text{cm}}^{3}}$	$D_{10}$	$D_{30}$	$D_{50}$	
$B_{\text{Full}}$	≤4	41.1±13.5	57.2±20.6	36.1±9.9	26.4±6.5	20.8±5.7	10.8
	>4	50.3±28.0	77.6±74.8	48.0±30.9	36.3±21.3	29.8±16.5	8.2
$B_{\text{Empty}}$	≤4	44.6±17.0	62.8±25.5	39.1±12.8	27.6±7.6	21.4±6.3	9.1
	>4	48.3±17.8	67.6±32.4	45.5±20.8	34.7±15.4	28.8±12.9	8.7
p‐value		0.654	0.679	0.626	0.572	0.448	0.631

a Min. Dis. is the mean of minimum distance between the organ and ${\text{CTV}}_{\text{HR}}$, calculated as $\Sigma P_{i}/n$; where $P_{i}$ is the distance and *n* is the number of samples

T.L.=tandem length

## IV. DISCUSSION

The impact of bladder volume on a number of DVH parameters of two series of treatment plans created on full and empty bladder CT images was assessed, prospectively. The study comprised of 20 patients selected for GYN brachytherapy treatment with Rotterdam tandem‐ovoid applicators. Tandem length was incorporated as an influencing factor on dose to OARs and its effect was investigated in both bladder conditions.

We tried to minimize interobserver discrepancies by having a single physician contour organs on both CT image series of full and empty bladder.^(15)^ Treatment planning was done in a 3D environment and timely procedures for reporting the results were used based on GEC‐ESTRO recommendations. The $D_{90}$ for ${\text{CTV}}_{\text{HR}}$ was kept almost constant between the two plans (maximum different was ∼2%).

Our findings agree with prior investigations.^5^, ^16^ In a study with 20 patients undergoing intracavitary treatment, bladder volume was not found to have a notable effect on rectal dose, but the median bladder wall was significantly affected.^(3)^ Increased bladder volume was also found to increase ICRU‐38 bladder base maximum dose point.^(4)^ Similar to these reports, OARs received higher dose in full bladder plans than empty bladder plans in our study (see Table 1). High dose parameters of organs at risk (i.e., $D_{2{\text{cm}}^{3}}$, $D_{1{\text{cm}}^{3}}$, and $D_{0.1{\text{cm}}^{3}}$) in the $B_{\text{Full}}$ group were surpassing those of the $B_{\text{Empty}}$ group.

In some other studies bladder distension was found to have no significant influence on bladder, rectum, and sigmoid doses.^6^, ^17^ This was contrary to what we observed in our study. The reason behind this might be methodological, as we compared the full bladder volume of a specific patient to her own empty bladder volume. In previous studies however, various bladder volumes of different patients were calculated and compared to one another; as such, additional patient specific features, such as OARs' wall elasticity or their relative distances, could potentially contribute to producing somewhat different results.^6^, ^17^

Longer tandem resulted in a larger dose to sigmoid in both bladder conditions which was more severe when the bladder was full. Therefore, when a longer tandem is used, treating with an empty bladder is likely to better preserve sigmoid (see Table 3). This did not result in a larger dose to bladder as $D_{2{\text{cm}}^{3}}$, $D_{1{\text{cm}}^{3}}$, and $D_{0.1{\text{cm}}^{3}}$ did not show significant variations with longer tandems in both bladder conditions. When T≤4 cm, empty bladder received a lower dose, but at the cost of a rectal dose increase (see Fig. 1 and Table 2). Therefore, the choice of whether to treat with a full or empty bladder when a shorter than 4 cm tandem is used depends on which organ may have a higher priority for the oncologist to preserve.

Senkus‐Konefka et al.^18^, ^19^ retrospectively studied the influence of intracavitary applicators on pelvic dose distribution during cervical cancer brachytherapy and found significant correlation between applicator size and the dose to bladder and rectum ICRU points, as well as the pelvic wall. Their results indicated lower dose in bladder and rectum and higher dose in ICRU point B with larger vaginal applicators, while longer tandems produced lower dose in rectum and higher dose in point B. They concluded that the use of larger applicators resulted in superior dose distribution and could be of benefit to the patient. In another study by this group, longer tandem length was reported to produce lower and higher dose to rectum and bladder ICRU points, respectively.^18^, ^19^ Our results agree with their findings. The only minor exception is the 3% higher rectum $D_{2{\text{cm}}^{3}}$ value we obtained for longer tandem length in $B_{\text{Full}}$ group (see Table 2).

In the literature it is suggested that the bladder should be kept full in all treatment fractions as it then pushes the intestine upwards and preserves the small bowel.^6^, ^16^ In clinical practice, some practitioners would deliberately employ a full bladder to displace the small bowel when it falls anterior to the uterus. According to our results, this is not always the best practice, considering other pelvic normal tissues at risk of radiation damage such as bladder, rectum, and sigmoid. In our case, filling the bladder increased high‐dose parameters to OARs (Table 1) which may induce treatment‐related complications, if not compensated in repeated fractions.

## V. CONCLUSIONS

It is clear that the planner tries various possibilities to reduce the dose to OARs when preparing a treatment plan for a specific patient. This is a highly individualized procedure for the patient at the time of treatment. A treatment team, however, may develop certain techniques, tips, and rules of thumb that can be useful in improving the outcome of their treatments in general. We conducted this study to evaluate the concurrent effect of bladder volume and tandem length, and to investigate whether we can come up with an improved bladder filling protocol for our treatments. Our results are currently limited to tandem angles of 30° and tend to suggest the followings.

For patients with tandem length longer than 4 cm, the bladder volume tends to have limited influence on bladder dose parameters. With an empty bladder, $D_{2{\text{cm}}^{3}}$, $D_{1{\text{cm}}^{3}}$, and $D_{0.1{\text{cm}}^{3}}$ dose metrics are slightly decreased (e.g., about 2% of planning aim dose for $D_{2{\text{cm}}^{3}}$), while low and intermediate dose parameters ($D_{10},D_{30}$, and $D_{50}$) are prone to higher doses. An empty bladder generally results in lower dose values to rectum and sigmoid; therefore, with a tandem length larger than 4 cm, a discharged bladder is likely to better preserve these organs.

Based on our results, in case of small tandem lengths (shorter than 4 cm), we tend to suggest a moderate bladder filling (e.g., injecting about 50–120 cm^3^ normal saline into the bladder) to reduce doses to the rectum, sigmoid, and small bowel. In such situations, it is often desirable to reduce dose to the rectum, sigmoid, and small bowel at the cost of a slightly higher dose to bladder.

We plan to extend this study to investigate potential effects of other tandem angles and other types of applicators on more dose parameters and treatment conditions of our GYN brachytherapy treatments.

## COPYRIGHT

This work is licensed under a Creative Commons Attribution 4.0 International License.

## Supporting information

Supplementary Material

Supplementary Material

Supplementary Material

Supplementary Material

Supplementary Material

Supplementary Material

Supplementary Material

Supplementary Material

Supplementary Material
