# Supplementary material for: A comparison of organs at risk doses in GYN intracavitary brachytherapy for different tandem lengths and bladder volumes
Source: J Appl Clin Med Phys. 2016 May 8;17(3):5–13. doi: 10.1120/jacmp.v17i3.5584 (PMC5690927; doi:10.1120/jacmp.v17i3.5584)
Supplement: Supplementary file 1 — Supplementary Material [file ACM2-17-005-s001.docx]

**A comparison of organs at risk dose in GYN intracavitary brachytherapy according to different tandem length and bladder volume**

**Abstract:** Background and Purpose: To investigate the tandem length and bladder volume effects on the pelvic OARs received dose, in HDR intracavitary brachytherapy of cervical cancer.

Material and Methods: Twenty insertions of locally advanced cervical cancer patients were selected. They had complete or partially response to 3D EBRT. Appropriate Rotterdam HDR Applicators were inserted and 2 CT scan were done with empty and full bladder condition. 3D treatment planning was done for each of the image series. Patients were classified in: T > 4cm and T ≤ 4cm groups, due to their tandem length. DVH parameters of OARs were analyzed.

Results: Simultaneously considering the bladder volume and tandem length effects on the bladder dose, led to the significant differences (P < 0.05) of D2cc, D1cc and D0.1cc, for full and empty groups, whom were treated by the T ≤ 4cm. For T > 4cm ones, calculated data discrepancies were not significant. In empty bladders, rectum dose decreased for T > 4cm ones. For T ≤ 4cm class, full bladder led to lesser rectum dose. For sigmoid, by increasing the tandem length, sigmoid dose increased.

Conclusions: For T > 4cm group, the bladder volume has limited influence on the bladder dose parameters. For the rectum and sigmoid doses an empty bladder condition showed lower dose values. For T ≤ 4 cm ones, It seems appropriate to consider a moderate bladder filling (e.g. injecting about 50-120cc normal saline to the bladder) to spare rectum, sigmoid and small bowel.

**Keywords:** tandem length, Cervical Cancer, brachytherapy

PACS number: 87.53.Jw

**I. Introduction**

For patients who suffer from locally advanced cervical cancer, brachytherapy is an essential component of radio-chemotherapy [1]. HDR brachytherapy with 3D treatment planning capability is becoming a widespread treatment modality for these patients.

3D treatment planning require the use of 3Dor reconstructed images like CT, MR as a basis for image-guided brachytherapy (IGBT). A “wide therapeutic window” is the aim of IGRT[2]. The anatomy and topography of the bladder has a substantial influence on bladder dose values [3-8]. Intrauterine tandem length selection depends on patients’ anatomy, especially uterus channel length and malignancy extension, selected by the clinical radiotherapy oncologist. The purpose of this study was to investigate the effects of tandem length and bladder volume on the pelvic OARs received treatment dose, for different bladder volumes.

In this study the volume of the bladder was controlled to beeitherempty or filled with 120 cm³ normal saline.Although, achieving a complete empty bladder was impossible, in this study it was attempted to take the bladder volume as low as possible.

The major advantage of this study is, that in contrast to previous investigations the bladder volume was controlled actively, while anatomical condition of all other pelvic OARs (i.e. rectum, sigmoid etc) were kept stable for two image series of each patient.

**II. Methods**

1. ***Case selection***

Twenty insertions of locally advanced cervical cancer patients were selected over a period of 18 months (i.e. from June 2012 until November 2013). Chosen patients had complete or partially response to 3D conformal EBRT (prescribed dose of 45-50Gy in five to six weeks duration). The patients also took concomitant Cisplatin-based chemotherapy of 50mg/m2 weekly. HDR intracavitary brachytherapy was prescribed after completion of EBRT + chemotherapy treatment. Patients had different FIGO (Federation of Gynecology and Obstetrics) stages of IB1, IB2 and IIB[9].

1. ***Applicator insertion***

Intracavitary Rotterdam titanium HDR tandem-ovoid applicators (Nucletron, the Netherlands now Elekta AB, Sweden) were inserted in a dedicated brachytherapy center (Brachytherapy Department, Atieh Hospital, Tehran, Iran) [10]. Available tandem lengths in this center were 3, 4, 5 and 7 cm. The only available tandem angle was 30°, which was fitting to the selected patient’s uterine properly. Used ovoid caps diameters were 2 and 3cm. The applicator insertion was done by the clinical radiotherapy oncologist in the operation room and under general or regional anesthesia. Foley catheter was inserted into the patient’s bladder. The bladder balloon was filled with 1cc of Meglumin compound mixed with 6cc of normal saline. After patient recovery and applicator fixation, a CT scan was performed.

1. ***Imaging***

Patients were placed on a flat board, on the CT scanner (Somatom DR, Siemens) table, in supine position. First of all 120cc of normal saline and some Meglumin compound (for better bladder delineation) were injected into the Foley catheter and its lumen was clamped. Then, a CT scan was done in this filled (full) bladder condition. Second, the clamp was opened and the patient was asked to empty her bladder as much as she can. After that, the CT scan was repeated with the emptied(empty) bladder condition. Thickness of CT slices were 3 mm for a whole pelvic scan. The repeated imaging process (i.e. in full and empty bladder condition) was performed just in one fraction of brachytherapy for each patient. Ethical rules considerations were followed and patients were informed about the imaging and therapy procedures.

1. ***Contouring and treatment planning***

Flexiplan (version 2.6, Isodose control, the Netherlands), a 3D HDR brachytherapy treatment planning system, was used. The calculation algorithm is based on TG-43[11]. Sectional imaging data were imported to this system. Flexiplan has the option of image reconstruction in sagittal and coronal series from the axial slices. For each of the patient intermediate-risk CTV (IR CTV) and high-risk CTV (HR CTV) contouring in two CT scan slices were done by the same clinical radiotherapy oncologist and on the basis of GEC-ESTRO recommendations[12-13].OARs contouring was also performed by oncologist or in corporation with a medical physicist. The OARs in this study were bladder, rectum, and sigmoid.

The reconstruction of the inserted applicators was done by choosing the tandem and ovoids from the applicators library of the treatment planning system and positioned to their correct location by the three point selection protocol.

For each series of images (i.e. empty and full bladder ones) treatment planning was done separately but, with the same applicator type and planning aim dose. Each clinical case was considered as an individual plan, where all dose constraints for OAR had to be fulfilled as close as possible. So, we had to pay penalty by decreasing D90s for HR CTVs. However, D90 was the primary parameter, which had to be equal as close as possible between the two bladder conditions. The amount of dose per fraction for brachytherapy was obtained by the usage of biologically equivalent dose in 2 Gy/fraction (EQD2) and linear-quadratic model with $\alpha/\beta=10$Gy for CTVs and $\alpha/\beta=3$Gy for OARs[2].GEC-ESTRO guidelines for target contouring and dose constrains for normal tissues and CTVs were considered in manually optimization with the Flexiplan system [12-14]. The treatment purpose was to achieve at least 80-90Gy total dose to D90 of the HR-CTV (EQD2). Maximum dose of 70Gy for D2cc rectum and 80Gy for D2cc bladder were defined as relevant dose constrains[13].

Iridium-192 was used as a HDR source and in combination with Flexitron remote afterloading brachytherapy delivery system.

The routine protocol for the treatment of the patients in the mentioned center was filling the patient bladder; by again 120 cc normal saline, and clamp it during the CT scan procedure. After taking the images the clamp was opened for patient's comfort. The bladder was filled again with the same volume of normal saline just before starting to load the iridium source to the applicators, in order to achieve the same situation of CTVs and OARs. The patients were treated with the standard protocol without variation. Only the DVH parameters of OARs and CTVs were recorded after applying the treatment planning for both of described bladder filling conditions of the patient.

1. ***Classification***

The main classification of this study was based on the bladder filling protocol as it was applied to the patients, prospectively. First of all, treatment planning data were classified in two groups: B-Full and B-Empty(B means bladder). On the other hand, tandem length effects on the OARs received dose for different fullness condition of these organs needs to be assess, too. So, patients who were treated with various tandem lengths were divided in two classes of: T > 4cm with fourteen cases and T ≤ 4cm with six cases.

1. ***Data analysis***

After obtaining the DVH parameters of applied treatment planning for each series of patient’s images, statistical tests, such as Paired Samples T-test, was performed on the data (with SPSS 17). Additionally, for taking the effect of tandem length in to account on the resulted DVH parameters, simultaneously, General Linear Model-Multivariate (GLM) test was also used. All the mentioned analysis was done by confidence intervals (CI) of 95.0% (significance level = 0.05).

**III. Results**

To be sure about the validity of our statistical results, normality of raw data distributions for both mentioned tests (with Kalmogorov-Smirnov test) and also homogeneity of the variances (with Levene's test) for GLM test were checked and passed the criteria. The HR-CTV volume differences were 0.3±2.1% showing no significant difference between the full and empty bladder condition image series. The D90 for HR-CTV in full bladder cases was systematically only 2% smaller compared to empty bladder cases without any significant difference. This is the proof that the treatment plans are based on the same dose prescribed to the D90 of the HR-CTV.

Mean ± SD of empty and full bladders’ volumes were 59.7±18.8cc and 177.4±44.6cc, respectively. On the other hand, because CTs were taken subsequently, with as minimum as possible time delay, it’s expected to have just a little discrepancy in sigmoid and rectum volumes of two series. Mean of these volume differences were also not significant (0.5±5.9% for rectum and 0.6±4.6% for sigmoid), which underlines this assumption.

***A-Effect of bladder volume on the OARs received dose***

Comparison of bladder, rectum and sigmoid DVH parameters, for two treatments plans with full and empty bladder condition is illustrated in Table 1. As it can be seen, differences in doses and volumes of the bladders are significant (P<0.05) (except for V100)between the two groups. For rectum and sigmoid, as expected, received doses for the full bladder condition are higher than the empty ones, but not significant. The results (Mean(full-empty)±SD) for EQD2 comparison for D2cc of bladder were 4.2±2.4 Gy EQD2 per fraction in case of tandem length ≤ 4 cm. Assuming 3-4 fraction this is related to a substantial dose decrease for empty bladder volumes of > 10 Gy EQD2 total dose.

***B-Effect of tandem length and bladder volume on the OARs received dose***

***B-1-Bladder***

The results for simultaneously considering the effects of bladder volume and inserted tandem length on bladder DVH parameters were shown in the Fig.1 and Fig.2. In these figures, significant differences (P<0.05) can be illustrated for D2cc, D0.1cc and D1cc of full and empty bladder groups, when treated using a tandem length of below or equal to 4cm. For tandem length longer than 4cm, discrepancies between the obtained results are not significant.

On the other hand, for other demonstrated DVH parameters differences are very limited between the full and empty conditions (Fig. 1). Dose to D10, D30, D50 and V50 of empty bladders are more than full ones, for cases that were treated with tandems longer that 4cm.Almost no difference in the mentioned doses can be seen between two groups (full and empty bladder), for tandem length shorter than 4cm.

***B-2-Rectum***

As it can be observed in Table 2, the effect of tandem length on the received dose by the rectum in full and empty bladder groups is not significant, but in empty condition dose to rectum decreased for tandem length longer that 4cm. For tandem length equal or shorter than 4cm full bladder led to lesser dose to rectum than the empty ones.

***B-3-Sigmoid***

For sigmoid, as presented in Table 3, differences was not found to be significant after statistical analyzes, in different classified groups. However, for longer tandem length, mean doses to the sigmoid were higher than for the shorter tandem lengths.

**IV. Discussion**

In a prospective study, impact of bladder volume on the obtained DVH parameters of treatment planning for two CT image series (B-Full and B-Empty) of twenty patients, whom were treated with intracavitary brachytherapy with tandem-ovoid applicators, were assessed. In addition the effect of the tandem length in case of full and empty bladder condition was studied. Contouring of both image series of each cases were done by a single Physician for elimination of the inter-observer discrepancies[15]. The effect of different bladder volumes on the various DVH parameters of bladder, rectum and sigmoid are the same as some of the previously research groups ones [5, 16]. Sun et al studied 20 intracavitary patients and found not any significant variation in the rectal dose by various bladder volumes. On the other hand, they concluded that bladder distension reduce the median bladder wall significantly[3].Adli et al. evaluated the treatment of 42 cases of intracavitary HDR. Their aim was to evaluate the bladder volume impact on the ICRU-38 Bladder Reference Point (BRP) and Bladder Base Maximum Point (BBMP) dose for orthogonal radiographs based intracavitary brachytherapy planning. They found that BBMP would increase by increasing bladder volume[4].

In the presented assessment, 3D treatment planning was done and up to dated procedure for reporting the results, which are based on GEC-ESTRO recommendations, were used. Furthermore, it had been tried to omit other variables, except bladder volume for each image series and anatomical condition. This research protocol is one of the results of some discrepancy between the obtained results by the other published works like GÖÇEN et al. ones. They found no difference at reference point’s dose by various bladder volumes of 61 distinctive brachytherapy cases and suggested making the bladder full for every brachytherapy fraction [17]. Patra et al. also studied 47 cervical cancer patients brachytherapy treatment and found that bladder distension has no any significant influence on the bladder, rectum and sigmoid[6].

In this case the D90 for HR-CTV was kept almost constant between the two plan versions (~2% different). The OARs in general received higher doses in full bladder conditions. From the presented study it can be concluded that by increasing the tandem length, dose to sigmoid will increase even for empty or full bladder, but for full bladder this effect of tandem length is more severe. So, by just having the sigmoid dose in mind, when one decide to treat a patient for whom the physician inserts a longer tandem, the empty bladder helps lesser dose delivery to sigmoid. The results that have been obtained for bladder also support this proposal, because as it was mentioned earlier (Fig. 1), dose to the bladder hot small volumes (i.e. D2cc, D0.1cc and D1cc) have not a lot of difference between B-Full and B-Empty groups, for longer tandem length insertion condition. However, for the cases with ≤ 4cm tandem length, due to the fact that in full bladder, dose to the bladder hot spots will increase but to the rectum and sigmoid will decrease (Fig.1 and Table 2 and 4), there is some controversy between having an empty or full bladder for the patients.

Senkus-Konefka et alstudied the influence of low-dose rate brachytherapy applicators in a retrospective study of different patients and reported that size of applicators can affect the quality of dose distribution to the bladder and rectum ICRU points and also pelvic wall. They conclude that larger intra-uterine applicators results in better dose distributions. In another study of this group they said that longer tandems will cause lower rectum and higher bladder ICRU point dose [18-19]. Their findings are to some extent in agreement to the presented investigation but not for the empty bladder condition (Table 2).

Some radiotherapy researchers also just pay attention to the small bowel and propose to have full bladder in any treatment fraction, for pushing the intestine above. Despite the fact that full bladder can push the bowel upside, but can have some adverse effects on the others pelvic normal tissues, by ignoring the different relative situation of bladder due to its volume and tandem length, which were investigated here[6, 16].

The final dose to OAR will mainly depend on the dose optimization possible for each individual patient. However, by selection of an appropriate filling protocol the dose to the bladder can be influenced in addition. For short tandem length (≤ 4 cm) it could be considered to completely empty the bladder to reduce the bladder dose. However, as this will result in higher rectum and sigmoid dose, care should be taken to control these doses individually.

**V. Conclusion**

The mutual influence of tandem length, bladder volume and OAR dose is highly individual, but some general rules for patient preparation can be drawn from these results.

For patients with tandem length >4cm the bladder volume has limited influence on the bladder dose parameters. For high dose volumes (D2cc, D1cc and D0.1cc) empty bladder conditions result in slightly smaller dose. In case of low and intermediate dose parameters (D10, D30, D50) increasing bladder volume is linked to lower doses. For the rectum and sigmoid doses an empty bladder condition showed lower dose values.

For patients with tandem length ≤ 4cm an empty bladder is clearly linked to lower bladder doses values. However, rectum and sigmoid doses are on average smaller for the full bladder condition. Therefore in case of small tandem length, no general rule can be applied. It seems appropriate to consider a moderate bladder filling (e.g. injecting about 50-120cc normal saline to the bladder) to spare rectum, sigmoid and small bowel.

**References**

[1] Halperin EC, Perez CA, Brady LW. Perez and Brady's principles and practice of radiation oncology. 5th ed. / edited by Edward C. Halperin. ed. Philadelphia, Pa. ; London: Wolters Kluwer/Lippincott Williams & Wilkins; 2008.

[2] Viswanathan AN, SpringerLink (Online service). Gynecologic radiation therapy novel approaches to image-guidance and management. Berlin, Heidelberg: Springer-Verlag Berlin Heidelberg; 2011. p. ix, 308 p.

[3] Sun LM, Huang HY, Huang EY, Wang CJ, Ko SF, Lin H, et al. A prospective study to assess the bladder distension effects on dosimetry in intracavitary brachytherapy of cervical cancer via computed tomography-assisted techniques. Radiother Oncol. 2005;77:77-82.

[4] Adli M, Garipagaoglu M, Kocak Z. Effect of bladder distention on bladder base dose in gynaecological intracavitary high dose rate brachytherapy. Br J Radiol. 2009;82:243-8.

[5] Cengiz M, Gurdalli S, Selek U, Yildiz F, Saglam Y, Ozyar E, et al. Effect of bladder distension on dose distribution of intracavitary brachytherapy for cervical cancer: three-dimensional computed tomography plan evaluation. Int J Radiat Oncol Biol Phys. 2008;70:464-8.

[6] Patra NB, Manir KS, Basu S, Goswami J, Kabasi AK, Sarkar SK. Effect of bladder distension on dosimetry of organs at risk in computer tomography based planning of high-dose-rate intracavitary brachytherapy for cervical cancer. J Contemp Brachytherapy. 2013;5:3-9.

[7] Pilepich MV, Prasad S, Madoc-Jones H, Bedwinek JM. Effect of bladder distension on dosimetry in gynecological implants. Radiology. 1981;140:516-8.

[8] Wyckoff HO, Allisy A, Caswell RS, Adams GED, Cowper G, Edholm P, et al. ICRU Report 38: Dose and Volume Specification for Reporting Intracavitary Therapy in Gynecology. 1985.

[9] Pecorelli S. Revised FIGO staging for carcinoma of the vulva, cervix, and endometrium. Int J Gynaecol Obstet. 2009;105:103-4.

[10] Cervix Rotterdam Applicator. wwwpmlfilestorecom/gms/pdfs/Cervix-Rotterdam-applicatorpdf‎.

[11] Rivard MJ, Coursey BM, DeWerd LA, Hanson WF, Huq MS, Ibbott GS, et al. Update of AAPM Task Group No. 43 Report: A revised AAPM protocol for brachytherapy dose calculations. 2005.

[12] Haie-Meder C, Potter R, Van Limbergen E, Briot E, De Brabandere M, Dimopoulos J, et al. Recommendations from Gynaecological (GYN) GEC-ESTRO Working Group (I): concepts and terms in 3D image based 3D treatment planning in cervix cancer brachytherapy with emphasis on MRI assessment of GTV and CTV. Radiother Oncol. 2005;74:235-45.

[13] Potter R, Haie-Meder C, Van Limbergen E, Barillot I, De Brabandere M, Dimopoulos J, et al. Recommendations from gynaecological (GYN) GEC ESTRO working group (II): concepts and terms in 3D image-based treatment planning in cervix cancer brachytherapy-3D dose volume parameters and aspects of 3D image-based anatomy, radiation physics, radiobiology. Radiother Oncol. 2006;78:67-77.

[14] Kirisits C, Potter R, Lang S, Dimopoulos J, Wachter-Gerstner N, Georg D. Dose and volume parameters for MRI-based treatment planning in intracavitary brachytherapy for cervical cancer. Int J Radiat Oncol Biol Phys. 2005;62:901-11.

[15] Dimopoulos JC, De Vos V, Berger D, Petric P, Dumas I, Kirisits C, et al. Inter-observer comparison of target delineation for MRI-assisted cervical cancer brachytherapy: application of the GYN GEC-ESTRO recommendations. Radiother Oncol. 2009;91:166-72.

[16] Kim RY, Shen S, Lin HY, Spencer SA, De Los Santos J. Effects of bladder distension on organs at risk in 3D image-based planning of intracavitary brachytherapy for cervical cancer. Int J Radiat Oncol Biol Phys. 2010;76:485-9.

[17] GÖÇEN F, ÖZSARAN Z, YALMAN D, ESASSOLAK M, OLACAK N, A H. THE INFLUENCE OF FULL BLADDER ON DOSE DISTRIBUTION DURING INTRACAVITARY BRACHYTHERAPY. Turk Onkoloji Dergisi. 2003;18:024-9.

[18] Senkus-Konefka E, Kobierska A, Jassem J, Badzio A. Patient-related factors determining geometry of intracavitary applicators and pelvic dose distribution during cervical cancer brachytherapy. Int J Radiat Oncol Biol Phys. 1997;37:531-6.

[19] Senkus-Konefka E, Kobierska A, Jassem J, Serkies K, Badzio A. Influence of brachytherapy applicators geometry on dose distribution in cervical cancer. Strahlenther Onkol. 1997;173:323-9.

**Figures**


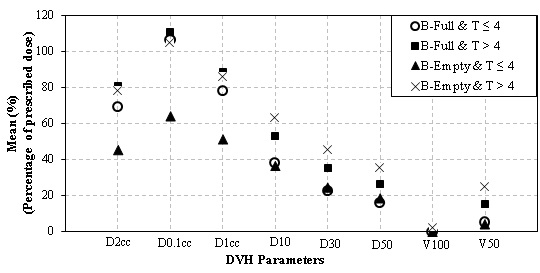


Fig.1.Result of bladder DVH parameters; comparison between two full and empty groups, by regarding the tandem length, simultaneously. (B: bladder, T: tandem length).


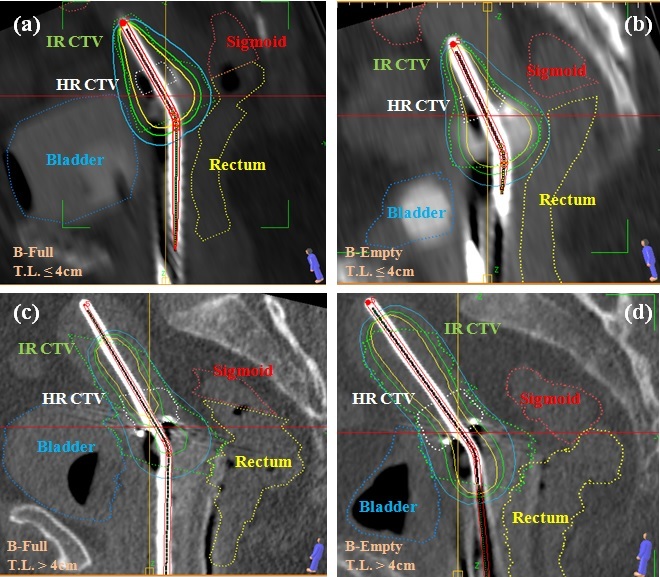


Fig. 2. Contoured CT scan examples of an applicator inserted cervical cancer patient. (a, b) tandem length ≤ 4cm and (c, d) tandem length > 4cm. (a, c) full bladder. (b, d) empty bladder.(T.L.: tandem length, B-Empty: empty bladder and B-Full.: full bladder).

**Tables**

Table 1. Paired samples statistical test results for comparison of OARs DVH parameters of full and empty bladder groups.

| Mean (difference of B^*^-Full & B-empty) DVH parameters (Percentage of prescribed dose)) ±SD  (P-value) | | | | | | | |  |
| --- | --- | --- | --- | --- | --- | --- | --- | --- |
| V50 | V100 | D50 | D30 | D10 | D_1_ cc | D_0.1_ cc | D_2_cc | Parameters |
| -6.7±11.2  (0.015) | -0.8±2.9  (0.232) | -7.1±7.6  (0.001) | -7.7±9.3  (0.001) | -6.6±12.6  (0.030) | 9.9±20.1  (0.047) | 17.1±31.1  (0.024) | 8.8±18.5  (0.047) | Bladder |
|  |  | 1.2±3.7  (0.193) | 0.7±5.0  (0.521) | 0.3±6.6  (0.853) |  | 2.0±13.4  (0.533) | 0.7±8.2  (0.726) | Rectum |
|  |  | 0.5±5.4  (0.707) | 0.8±7.3  (0.641) | 0.8±11.2  (0.744) |  | 5.3±38.4  (0.543) | 0.3±11.5  (0.901) | Sigmoid |

*: Bladder

Table 2. Multivariate statistical test results for comparison of rectum received dose in full and empty bladder groups and different tandem length.

|  |  |  |  | Mean of DVH parameters (Percentage of prescribed dose)) ±SD | | | | |
| --- | --- | --- | --- | --- | --- | --- | --- | --- |
|  |  | T. L.^*^ (cm) |  | D_2_cc | D_0.1_cc | D10 | D30 | D50 |
| Full B. |  | ≤ 4 |  | 66.6±6.9 | 90.5±13.2 | 56.9±9.5 | 39.4±8.4 | 30.4±7.1 |
|  |  | > 4 |  | 68.7±8.1 | 93.4±11.9 | 56.1±7.3 | 39.6±6.2 | 31.0±5.6 |
| Empty B. |  | ≤ 4 |  | 72.1±6.3 | 97.1±8.8 | 61.0±9.2 | 41.1±8.8 | 30.2±7.7 |
|  |  | > 4 |  | 66.5±9.2 | 89.3±13.5 | 54.7±6.8 | 38.4±6.1 | 29.9±5.5 |
| Sig. |  |  |  | 0.186 | 0.277 | 0.171 | 0.516 | 0.904 |

T. L.^*^: Tandem length.

Table 3. Multivariate statistical test results for comparison of sigmoid received dose in full and empty bladder groups and different tandem length.

|  |  |  |  | Mean of DVH parameters (Percentage of prescribed dose)) ±SD | | | | |
| --- | --- | --- | --- | --- | --- | --- | --- | --- |
|  |  | T. L.^*^ (cm) |  | D_2_cc | D_0.1_cc | D10 | D30 | D50 |
| Full B. |  | ≤ 4 |  | 41.1±13.5 | 57.2±20.6 | 36.1±9.9 | 26.4±6.5 | 20.8±5.7 |
|  |  | > 4 |  | 50.3±28.0 | 77.6±74.8 | 48.0±30.9 | 36.3±21.3 | 29.8±16.5 |
| Empty B. |  | ≤ 4 |  | 44.6±17.0 | 62.8±25.5 | 39.1±12.8 | 27.6±7.6 | 21.4±6.3 |
|  |  | > 4 |  | 48.3±17.8 | 67.6±32.4 | 45.5±20.8 | 34.7±15.4 | 28.8±12.9 |
| Sig. |  |  |  | 0.654 | 0.679 | 0.626 | 0.572 | 0.448 |

T. L.^*^: Tandem length.
