# Supplementary material for: A comparison of organs at risk doses in GYN intracavitary brachytherapy for different tandem lengths and bladder volumes
Source: J Appl Clin Med Phys. 2016 May 8;17(3):5–13. doi: 10.1120/jacmp.v17i3.5584 (PMC5690927; doi:10.1120/jacmp.v17i3.5584)
Supplement: Supplementary file 2 — Supplementary Material [file ACM2-17-005-s002.docx]

**A comparison of organs at risk dose in GYN intracavitary brachytherapy according to different tandem length and bladder volume**

**Zahar Siavashpour^1*^, Mahmoud Reza Aghamiri^1^, Ramin Jaberi^2^, Naser ZareAkha^3^, Hamid Reza Dehghan Manshadi^4^, Christian Kirisits^5^**

*1-Department of Medical Radiation Engineering, Shahid Beheshti University, Tehran, Islamic Republic of Iran,* [*zahrasiavashpour@gmail.com*](mailto:zahrasiavashpour@gmail.com) *& z_siavashpour@sbu.ac.ir*

*2-Department of Radiotherapy, Tehran University of Medical Science, Tehran, Iran Islamic Republic of,* [*rjaberi@tums.ac.ir*](mailto:rjaberi@tums.ac.ir)

*3- Department of Brachytherapy, Pars Hospital, Tehran, Iran Islamic Republic of,*

*4- Department of Radiotherapy, Hafte Tir Hospital, Tehran, Iran Islamic Republic of*

*5- Department of Radiotherapy and Oncology, Comprehensive Cancer Center, Medical University of Vienna, Austria, christian.kirisits@meduniwien.ac.at*

**Corresponding author:** Zahra Siavashpour

*Department of Medical Radiation Engineering, Shahid Beheshti University,*

*Daneshjou BLV, Velenjak, Tehran, Islamic*

*z_siavashpour@sbu.ac.ir*

**A suggested running title:** Brachytherapy OAR dose dependency to tandem length and bladder volume
