# Supplementary figures and images for: A comparison of organs at risk doses in GYN intracavitary brachytherapy for different tandem lengths and bladder volumes
Source: J Appl Clin Med Phys. 2016 May 8;17(3):5–13. doi: 10.1120/jacmp.v17i3.5584 (PMC5690927; doi:10.1120/jacmp.v17i3.5584)

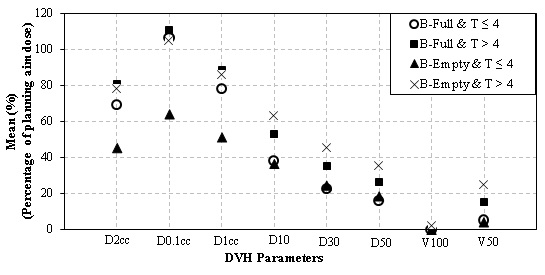

Supplement: Supplementary file 3 — Supplementary Material [file ACM2-17-005-s003.jpg]

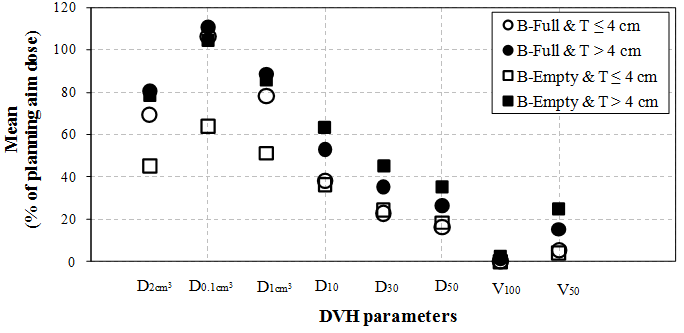

Supplement: Supplementary file 4 — Supplementary Material [file ACM2-17-005-s004.png]

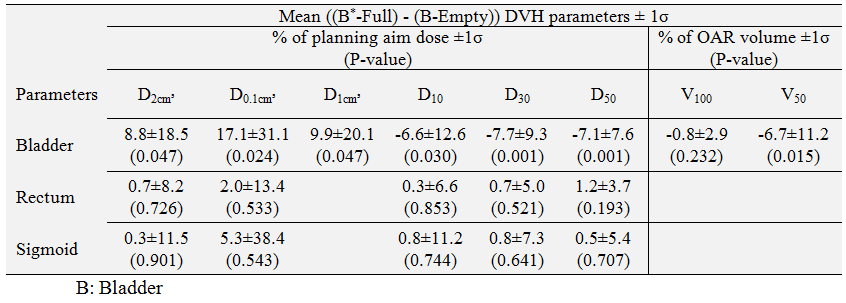

Supplement: Supplementary file 5 — Supplementary Material [file ACM2-17-005-s005.png]

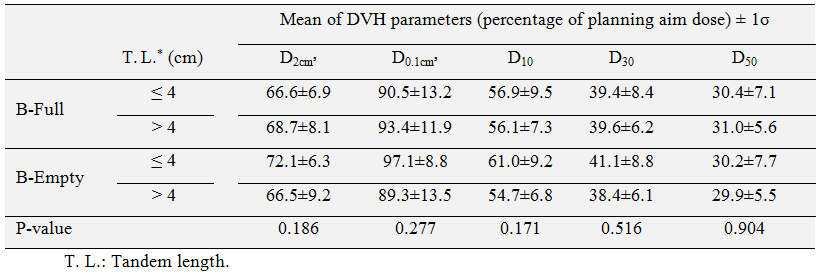

Supplement: Supplementary file 6 — Supplementary Material [file ACM2-17-005-s006.png]

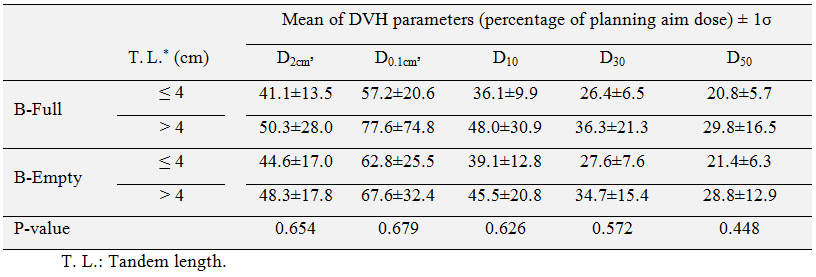

Supplement: Supplementary file 7 — Supplementary Material [file ACM2-17-005-s007.png]
