# Supplementary material for: A comparison of organs at risk doses in GYN intracavitary brachytherapy for different tandem lengths and bladder volumes
Source: J Appl Clin Med Phys. 2016 May 8;17(3):5–13. doi: 10.1120/jacmp.v17i3.5584 (PMC5690927; doi:10.1120/jacmp.v17i3.5584)
Supplement: Supplementary file 8 — Supplementary Material [file ACM2-17-005-s008.docx]

**A comparison of organs at risk doses in GYN intracavitary brachytherapy for different tandem lengths and bladder volumes**

**Abstract:**

**Background and Purpose:** To investigate the concurrent effects of tandem length and bladder volume on dose to pelvic organs at risk (OARs) in HDR intracavitary brachytherapy treatment of cervical cancer.

**Materials and Methods:** Twenty patients with locally advanced cervical cancer were selected for brachytherapy (BT). They had complete or partial response to 3D external radiotherapy (EBRT). BT was performed using appropriate Rotterdam applicators. The patients were CT scanned twice with empty and full bladder. Two treatment plans were prepared on each of the image sets. Patients were categorized into two groups; those treated with a tandem length of 4 cm or smaller (T ≤ 4 cm) and those with tandem length larger than 4 cm (T > 4 cm). Dose volume histograms (DVHs) of OARs were calculated and compared.

**Results:** Bladder dose was significantly affected by both bladder volume and tandem physical length for T ≤ 4 cm. This was reflected on the values obtained for D_2cm³_, D_1cm³_, and D_0.1cm³_ for both empty and full bladder cases. When T > 4 cm no correlation could be established between variations in bladder dose and bladder volume. Rectum dose was generally lower when the bladder was empty and T > 4 cm. Dose to sigmoid was increased when T > 4 cm.

**Conclusions:** Our results suggest that should a tandem of longer than 4 cm be used in GYN brachytherapy, keeping the bladder empty may reduce the dose to rectum and sigmoid. This is contrary to cases where a shorter than 4 cm tandem is used in which a full bladder (about 50-120 cm³) tends to result in a lower dose to rectum and sigmoid. Attention should be given to doses to sigmoid with long tandem lengths as a larger tandem generally results in a larger dose to sigmoid.
